# In Silico Analyses and Cytotoxicity Study of Asiaticoside and Asiatic Acid from Malaysian Plant as Potential mTOR Inhibitors

**DOI:** 10.3390/molecules25173991

**Published:** 2020-09-02

**Authors:** Ninie Nadia Zulkipli, Rahimah Zakaria, Idris Long, Siti Fadilah Abdullah, Erma Fatiha Muhammad, Habibah A. Wahab, Teguh Haryo Sasongko

**Affiliations:** 1 School of Medical Sciences, Universiti Sains Malaysia, Kubang Kerian 16150, Malaysia; nadiazulkipli93@yahoo.com; 2 School of Health Sciences, Universiti Sains Malaysia, Kubang Kerian 16150, Malaysia; idriskk@usm.my; 3 School of Dental Sciences, Universiti Sains Malaysia, Kubang Kerian 16150, Malaysia; sfadilah@usm.my; 4 School of Pharmaceutical Sciences, Universiti Sains Malaysia, Pulau Pinang 11800, Malaysia; ermafatiha@gmail.com; 5 School of Medicine, Perdana University-RCSI, Jalan MAEPS Perdana, Serdang 43400, Malaysia; teguh.haryo@perdanauniversity.edu.my

**Keywords:** mammalian target of rapamycin (mTOR), molecular docking, everolimus, asiaticoside, asiatic acid, IC_50_

## Abstract

Natural products remain a popular alternative treatment for many ailments in various countries. This study aimed to screen for potential mammalian target of rapamycin (mTOR) inhibitors from Malaysian natural substance, using the Natural Product Discovery database, and to determine the IC_50_ of the selected mTOR inhibitors against UMB1949 cell line. The crystallographic structure of the molecular target (mTOR) was obtained from Protein Data Bank, with Protein Data Bank (PDB) ID: 4DRI. Everolimus, an mTOR inhibitor, was used as a standard compound for the comparative analysis. Computational docking approach was performed, using AutoDock Vina (screening) and AutoDock 4.2.6 (analysis). Based on our analysis, asiaticoside and its derivative, asiatic acid, both from *Centella asiatica*, revealed optimum-binding affinities with mTOR that were comparable to our standard compound. The effect of asiaticoside and asiatic acid on mTOR inhibition was validated with UMB1949 cell line, and their IC_50_ values were 300 and 60 µM, respectively, compared to everolimus (29.5 µM). Interestingly, this is the first study of asiaticoside and asiatic acid against tuberous sclerosis complex (TSC) disease model by targeting mTOR. These results, coupled with our in silico findings, should prompt further studies, to clarify the mode of action, safety, and efficacy of these compounds as mTOR inhibitors.

## 1. Introduction

Tuberous sclerosis, also known as tuberous sclerosis complex (TSC) (OMIM 191100), is an autosomal dominant inherited disorder characterized by the presence of hamartomas (tumor-like lesions) in multiple organ systems, including the brain, kidney, heart, eyes, and skin [1]. TSC affects both sexes and multiple ethnic groups [1]. Its incidence and prevalence were estimated to be 1/10,000 live births and 1 in 6000 to 9000 individuals, respectively, and globally, at least two million people are affected [2]. TSC is due to the inactivating mutation in one of the TSC genes (*TSC1* or *TSC2*). These TSC genes are tumor-suppressor genes [3] and play the role as a GTPase-activating protein (GAP) toward Rheb, which is a major regulator of the mammalian target of rapamycin (mTOR). Dysfunction of TSC1 or TSC2 leads to the high levels of Rheb-GTP which cause constitutive activation of mTOR–raptor signaling, and thus promote the deregulating of protein synthesis and cell growth [4].

mTOR is a serine/threonine kinase that acts via two large and functionally distinct multiprotein complexes. The two complexes are mTOR complex 1 (mTORC1) and mTOR complex 2 (mTORC2). They sense and integrate multiple intracellular and environmental signals [5,6] and are implicated in many physiological functions [6]. In general, mTOR plays major roles in protein synthesis, a molecular sensor of gene transcription, oxidative stress, and immunity, as well as cell proliferation/cell death upon environmental and cellular cues [5,6,7]. More importantly, mTOR is one of the critical signaling hubs that are responsible for cancer cell growth [8] and ageing [9,10,11].

mTOR inhibitors can be classified into three generations [12]. The first generation mTOR inhibitors include rapamycin and the rapalogs, namely temsirolimus (CCI-779), everolimus (RAD001), ridaforolimus and Nab-rapamycin. Rapamycin has limited bioavailability; hence, it leads to the development of its rapalogs. In addition, rapalogs also have their limitations; for example, in terms of desired molecular effects, the efficacy may be partially limited by their drug action (cytostatic rather than cytotoxic) [13]. Besides, their use is limited to a few rare cancer types and not in the majority of solid tumors [14].

The most frequent adverse effects (AEs) associated with the mTOR inhibitors reported in patients include stomatitis, skin rash, and metabolic abnormalities (hyperlipidemia and hyperglycemia) [15,16,17,18]. Discontinuation of mTOR inhibitors treatment leads to tumor regrowth (such as kidney AML, SEGA, skin lesions, and cardiac rhabdomyoma) in the majority of patients [19]. Moreover, withdrawal from everolimus (prescribed for growing subependymal giant cell astrocytomas) led to relapse seizure twice, and the seizures were able to be controlled after reintroducing medicine [20].

**Figure 1:**
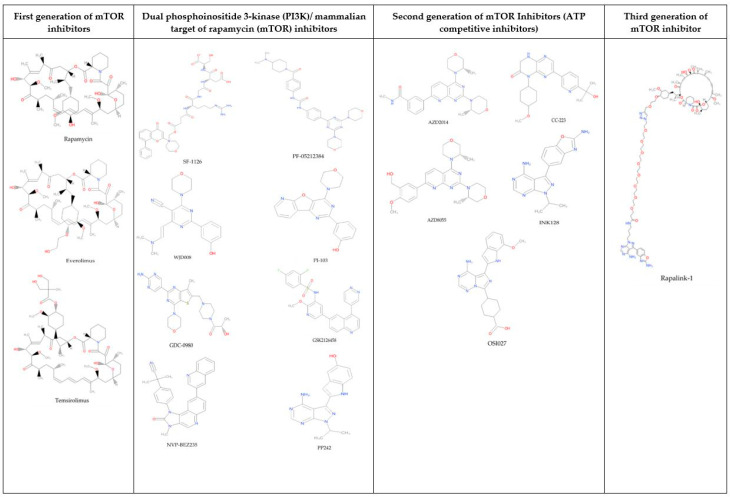
The chemical structures of the three generations of mTOR inhibitors and dual PI3K/mTOR inhibitors.

The dual PI3K/mTOR inhibitors, such as NVP-BEZ235, GSK2126458, XL765, and SF1126, are introduced, since they are capable of inhibiting two vital signaling hubs (PI3K and mTOR) that stimulate the growth of cancer cells. However, they still possess several disadvantages, such as in vitro resistance, limited efficacy in vivo [21,22], and critical toxicity, such as renal failure, hypertension [6], and elevated liver enzyme level (3 alanine aminotransferase (ALT) and asparate aminotransferase (AST)) [23,24]. Due to the weaknesses of the first-generation mTOR inhibitors and dual PI3K/mTOR inhibitors, the second-generation mTOR inhibitors, such as AZD8055, OSI-027, MLN0128, Torin 1, and AZD2014, were developed. However, the second-generation mTOR inhibitors caused several feedback loops that may trigger cancer cell survival and metastasis [25]. In addition, their effectiveness was reduced due to increased catalytic activity of mTOR following mTOR mutations [26,27]. Furthermore, they are more toxic to islet cells, compared to rapamycin [28]. To overcome all the weaknesses of the first- and second-generation mTOR inhibitors, the third-generation mTOR inhibitor known as RapaLink was developed. This third-generation mTOR inhibitor combined the properties of the first and the second generation of mTOR kinase inhibitors (TORKis) in the same molecule [27]. However, the development of this generation is still in the early stage, and further preclinical studies are required to fully understand its immunosuppressive properties [13]. Figure 1 shows the chemical structures of the three generations of mTOR inhibitors and dual PI3K/mTOR inhibitors.

Universiti Sains Malaysia has developed a resource database of potential compounds, collectively known as Natural Product Discovery (NADI©) (http://www.nadi-discovery.com). This online resource was developed to capture the plethora of information about Malaysia’s medicinal plants. It consists of several databases, such as NADI-Meps, NADI-Expert, NADI-Pub, and NADI-Visage, but the two most important are NADI-Herbs (collection of information of ethnopharmacological use of the plants) and NADI-Chem (a 3D Chemical Structure Database which contains more than 4000 compounds from over 361 plants) in a format amenable to virtual screening. NADI’s screening has been applied in various drug-discovery projects, including for ethno-scientific correlation of anti-tuberculosis drug discovery [29] and plants identification, such as for natural compounds screening and design of H5N1/H1N1 influenza’s neuraminidase inhibitors [30], the synthesis of potential new H1N1 neuraminidase inhibitors from ferulic acid and vanillin [31], and screening the potential inhibitors of isocitrate lyase for mycobacterium tuberculosis [32]. Here, we present our findings of potential mTOR inhibitors from NADI database and the cytotoxicity of the selected compounds against UMB1949 cell line.

## 2. Results and Discussion

To date, there are no standard drugs that can effectively and completely cure TSC. Even though everolimus has been used for years in treating TSC, unfortunately, there is still debate about the benefits and drawbacks. Natural products are a popular alternative for the treatment, due to a few factors, such as affordability [33,34], easy access by the community [33], their being mostly non-toxic, and their having minimum side effects [35], and with the advancement of science and technology, good efficacy, quality, and safety can be achieved [36].

Molecular docking is a computational approach to identify possible binding modes of the selected compound against its biological target [37]. This approach is possible between protein–ligand, protein–nucleotide, and protein–protein [38]. In our study, over 4000 compounds from the NADI database were virtually screened by AutoDock Vina. All the top 100 compounds possessed similar binding to the allosteric site as rapamycin in both FRB and FKBP5 residues (L2031, F2039, Y2105, F2108; Y57, F67, F77, V86, I87, W90, and I22). The top 100 compounds were derived from *Endiandra kingiana*, *Calophyllum inophyllum*, *Boesenbergia rotunda*, *Centella asiatica*, *Manilkara zapota*, and *Psidium guajava* and were reported to have a lower docking score than everolimus docking structure (−11.86 kcal/mol). The range of docking scores for the top 100 compounds was between 14.6 and −11.9 kcal/mol (Supplementary Materials Table S1).

None of the top 100 compounds shared the same physicochemical properties as everolimus. Thus, asiaticoside from *Centella asiatica* was selected from the top 100 compounds because it has lower docking score and an equal/lower Lipinski rules violation than everolimus. Asiaticoside successfully showed its antitumor activity in vitro (KM3/BTZ multiple myeloma cell line and human breast cancer (MCF-7) cell line [39,40] and in vivo (7,12-Dimethylbenz(a)anthracene (DMBA)-induced rat mammary cancer) [40]. Despite the higher ∆G value of asiatic acid (−11.54 kcal/mol) compared to everolimus, it was selected as a potential mTOR inhibitor due to its well-proven antitumor activity against various other types of cancer, such as human ovarian cancer, hepatoma, colon cancer, and breast cancer [41,42,43,44]. In addition, asiaticoside and asiatic acid have been proven to effectively improve memory [45,46,47] and ageing [48,49,50].

Natural compounds also possess the ability to retain low hydrophobicity and intermolecular H-bond donating potential as biologically active compounds with large numbers of rotatable bonds and high molecular weight [51]. For example, asiaticoside was reported to have lower LogP value when compared to everolimus (Supplementary Materials Table S2). This means that asiaticoside has higher solubility compared to everolimus. Solubility is a vital factor for absorption and can influence the bioavailability of a drug in vivo and also a significant element in lead generation and optimization [52]. Furthermore, natural products are more prone to resemble biosynthetic intermediates or endogenous metabolites than purely synthetic compounds and, thus, make use of active transport mechanisms [51]. Indeed, further justifications for selection of these compounds (asiaticoside and asiatic acid) as potential mTOR inhibitors are due to their relative lack of systemic toxicity [40,53,54], easily availability, and affordability.

**Figure 2:**
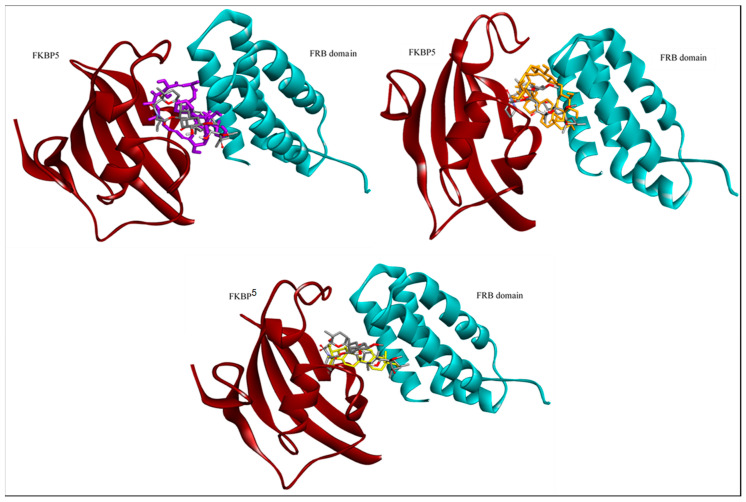
Binding poses comparison between crystallographic structure (rapamycin; gray color) and the lowest docking score for poses of everolimus (purple color), asiaticoside (orange color), and asiatic acid (yellow color), respectively.

Docking of the three selected compounds was performed, using AutoDock 4.2.6, by employing protein structure (FKBP5: FRB domain) and rapamycin to establish the validation of docking protocol. The binding modes and interaction for each complex were analyzed and viewed, using Accelrys Discovery Studio (Figure 2). The calculated docking scores for everolimus (positive control), asiaticoside, and asiatic acid were −11.86, −11.98, and −10. 37 kcal/mol, respectively (Table 1). Asiaticoside displayed relatively good binding affinity comparable to everolimus. Figure 2 shows the binding similarity between crystallographic structure (rapamycin bound complex) and the lowest docking score for pose of each compound. As depicted in Figure 2, everolimus does not directly bind to the FRB domain of mTOR protein rather than directly to FKBP5, which then blocks the access to the mTOR kinase active site. This site is located in a deep cleft and hydrophobic pocket behind the binding domain. Our control docking is also validated with a similar binding site which has been reported earlier [55]. It is suggested that the shared macrolide structure between rapamycin and everolimus permits interaction with FKBP5, subsequently to be selective to inhibit mTORC1 over mTORC2 [56]. It was observed that both of these triterpenoid compounds (asiaticoside and asiatic acid) bound at the similar binding region as everolimus, indicating a similar potential for the mTOR kinase inhibition activity (Figure 2).

**Figure 3:**
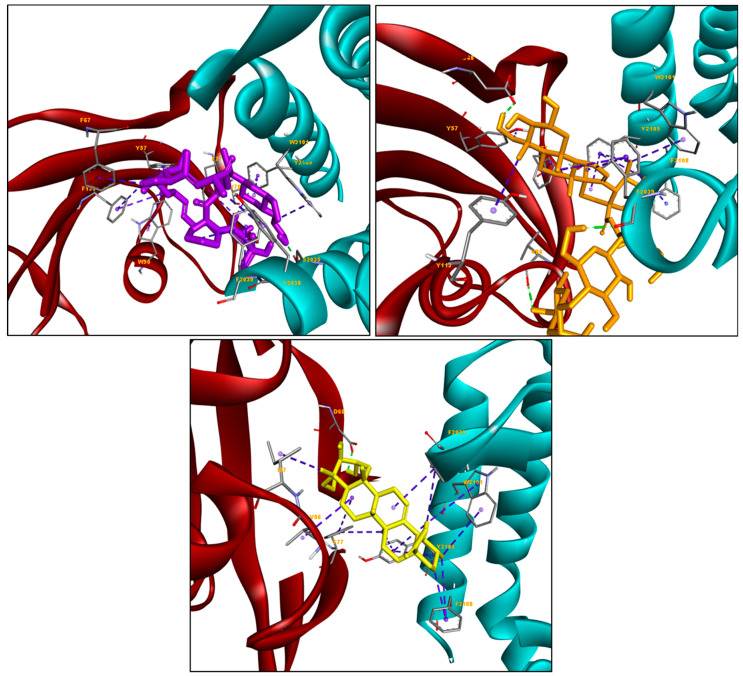
Showing the formation of the hydrogen bond(s) and hydrophobic interaction(s) between the amino acid residue in the active site of FKBP5/FRB human protein and each ligand. The green dotted line indicates the formation of the hydrogen bond(s), while the blue dotted line indicates the formation of the hydrophobic interaction(s).

Although these compounds were bound at the same binding mode, they showed a slightly different binding interaction. Based on Figure 3, we see the hydrophobic interactions are more commonly formed between the everolimus and hydrophobic residues (Phe78, Tyr57, Phe67, Phe77, Trp90, Phe130, and Trp2101 in FKBP5 domain; Tyr2038, Phe2039, and Tyr2105 in FRB domain), as compared to the hydrogen bond. For asiaticoside, the hydroxyl group of its substituent was found to interact with polar residues (D68 and F2039 in FKBP5 and FRB domains, respectively), while the aromatic rings of the compound were found to be buried in the hydrophobic sites containing residues Tyr57, Phe67, Phe77, Val86, Ile87, Tyr113, and Phe2039 in FKBP5; and Trp2101, Tyr2105, and Phe2108 in FRB. Meanwhile, asiatic acid’s hydrogen bond was formed with the D68 of FKBP5 residue, and intact more with hydrophobic residues (Tyr57, Phe77, Ile87, Val86, and Tyr113 in FKBP5; and Phe2039, Trp2101, Tyr2104, Tyr2105, and Phe2108 in FRB). With the selective interactions that occur between asiatic acid and the FKBP5/FRB domain, hydrophobic interactions may significantly influence the binding affinity of both triterpenoid compounds with FKBP5/FRB human protein.

**Figure 4:**
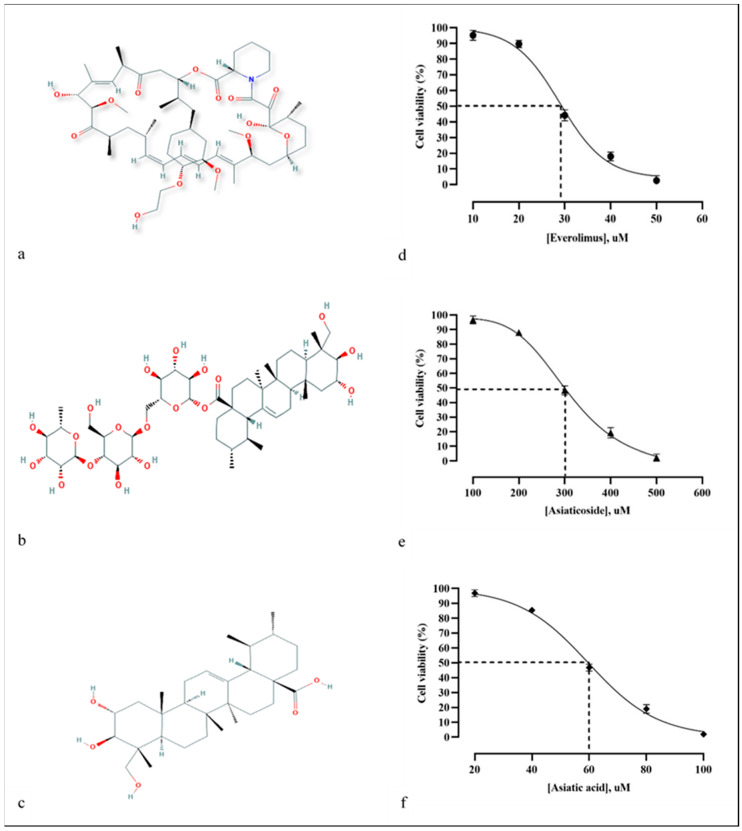
(**a**–**c**) Chemical structure of everolimus, asiaticoside, and asiatic acid, respectively. (**d**–**f**) The everolimus, asiaticoside, and asiatic acid depicted antiproliferative effect on the UMB 1949 cell line, as depicted by CCK-8 assay, respectively.

The effects on mTOR inhibition were validated by the bioassay approach, but the actual target validation was not carried out. The UMB1949 cell line was treated with different concentrations of everolimus, i.e., 10, 20, 30, 40, and 50 µM, for 24 h. The cytotoxicity of everolimus (Figure 4a) was dose-dependent, showing the highest cell viability (95.23% ± 3.11) at 10 µM and the lowest cell viability (2.67% ± 3.06) at 50 µM. The IC_50_ value of everolimus against the UMB1949 cell line was 29.5 µM (Figure 4d). The cytotoxicity of asiaticoside (Figure 4b) was also dose-dependent, with the highest cell viability (96.7% ± 2.71) at 100 µM and the lowest cell viability (2.33% ± 2.52) at 500 µM. The IC_50_ of asiaticoside against the UMB1949 cell line was 300 µM (Figure 4e). Similarly, asiatic acid (Figure 4c) also showed itself to be dose-dependent with the IC_50_ of 60 µM (Figure 4f).

**Figure 5:**
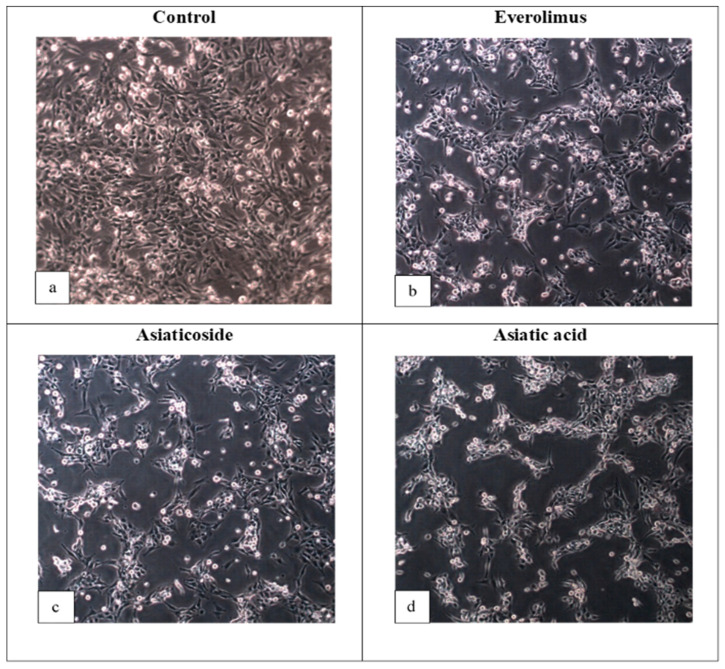
Photomicrographs of UMB1949 cell line (**a**) control-treated for 24 h with DMSO, (**b**) 29.5 µM everolimus, (**c**) 300 µM asiaticoside, and (**d**) 60 µM asiatic acid. Inhibition of cell growth was observed in treated samples (10× magnification).

The IC_50_ values of both compounds derived from *C. asiatica* were higher than everolimus, indicating that they are less cytotoxic (and also less potent) than everolimus. Interestingly, even though the docking score of asiaticoside was lower than that of everolimus, asiaticoside’s IC_50_ value was almost ten times higher than everolimus’s. This condition could be due to the physicochemical properties of the compounds which affect the penetration into cells, as shown by the higher log *p* of everolimus, when compared to asiaticoside (Supplementary Materials Table S2). This shows the lipophilicity of everolimus; thus, it more easily passes through the cell membrane (lipophilic barrier). Moreover, asiaticoside can be hydrolyzed to asiatic acid in vivo [57,58,59]. Asiatic acid, however, showed promising inhibitory activity with the IC_50_ of 60 µM, as compared to everolimus (29.5 µM). To the best of our knowledge, these are the first reported IC_50_ values for everolimus, asiaticoside, and asiatic acid against the UMB1949 cell line; thus, we were unable to make a comparison. Thus, the antiproliferative effect of these compounds was also determined (Figure 5). Everolimus exhibited promising results as an antitumor agent against the UMB1949 cell line (Figure 5b). Previously, everolimus has been used to treat several cancer types, such as advanced renal cell carcinoma, sub-ependymal giant cell astrocytoma (SEGA) associated with tuberous sclerosis, and advanced-hormone-positive and HER-negative breast cancer in combination with exemestane [60]. More importantly, asiaticoside and asiatic acid have successfully manifested their antitumor properties against the UMB1949 cell line (Figure 5c,d).

The antitumor drugs used previously are not only harmful to tumor cells but also the normal cells, and this restricts their clinical use [61]. Because of favorable safety and efficacy, natural compounds offer a choice over synthetic compounds [62]. Many phytochemicals have shown to possess antitumor, chemopreventive, or chemosensitizer effects, or act as adjuvants in attenuating adverse effects caused by chemotherapeutic drugs in cancer treatment [63]. Both asiaticoside and asiatic acid are from *C. asiatica*, a medicinal plant used in Malaysia and other parts of Asia for hundreds of years [64]. It is commonly known as *pegaga* in Malaysia, *mandukaparni* in India (Sanskrit), and *gotu kola* in Sri Lanka and China. *C. asiatica* was commonly used in the herbal nutraceutical industry and showed good efficacy, performance, and safety [65]. Most of its medicinal values are attributed to the presence of several triterpenes, namely asiatic acid, madecassic acid, asiaticoside, and madecassoside [66,67]. The constituents of *C. asiatica*, specifically asiaticoside, depict the great pharmacological effects as antibacterial [68], antioxidant [69,70], anti-ulcerative [71], anti-inflammatory [72], anxiolytic [73], anti-hepatofibrotic [74], and antidiarrheal agents, as well as for the treatment of asthma, tuberculosis, various skin lesions, wound healing [75,76], atherosclerosis, fungicidal and mental disorders [77], and ageing [48,49,50]. Moreover, asiaticoside 1 g/kg body weight has not shown to be harmful, and patients have shown good tolerance to *C. asiatica* extracts or asiaticoside [78]. Asiaticoside is the most abundant triterpene glycoside in the water extract, and it is transformed into asiatic acid in vivo by hydrolysis [79].

## 3. Materials and Methods

### 3.1. Accession of the Target Protein

The crystal structure of mTOR was retrieved from Protein Data Bank (www.rcsb.org), with Protein Data Bank (PDB) ID 4DRI [80]. This is a co-crystal structure peptidylprolyl isomerase (PPIase) domain of FKBP5 and FRB of mTOR of human protein bound with rapamycin. The atoms of rapamycin were discarded, and the amino acid structures, which were incomplete or have alternate conformers, were cleaned up by using Accelrys Discovery Studio [81]. Furthermore, AutoDock Tools [82] was used to add the hydrogens, to calculate charges, and to merge the non-polar hydrogens of protein structure. Next, the protein file was saved in PDBQT format.

### 3.2. Ligand Selection

The three-dimensional (3D) structures of Malaysian natural compounds and everolimus were obtained from the Natural Product Discovery (NADI) database (www.nadi-discovery.com) and DrugBank database (accession number: DB01590; https://www.drugbank.ca/drugs/DB01590), respectively. A Lipinski-like filter was applied before the PDB format was converted to PDBQT format, using Raccoon utility [83].

### 3.3. Analysis of Target Active Binding Sites

Accelrys Discovery Studio [81] was used to analyze and view their binding modes and the interactions between the active site of receptor and ligands.

### 3.4. Control Docking

Before performing the virtual screen of the NADI database, using AutoDock Vina, validation of our docking procedure was done by redocking rapamycin into the mTOR crystal structure (PDB: 4DRI) [80]. The structure of rapamycin in the co-crystal structure was separated from the proteins and was processed in a similar way as with everolimus. The docking’s searching space, represented by a grid box, was set at 35.461, 48.585, and 35.327 Å in x, y, and z coordinates, respectively (the center of mass of rapamycin), and the size of grid box was 58 × 40 × 40 points, with a spacing of 0.375 Å, to fit all the active site of rapamycin. Everolimus (positive control) was chosen as a benchmark in the virtual screening. Here we redocked everolimus at the same binding site of rapamycin, where the rapamycin binding sites in both FRB and FKBP5 are mostly hydrophobic (Leu2031, Phe2039, Tyr2105, Phe2108; Tyr57, Phe67, Phe77, Val86, Ile87, Trp90, and Ile22, respectively). This procedure was performed to ensure everolimus was successfully bound at the same active site of mTOR as by rapamycin.

### 3.5. Virtual Screening

Virtual screening was carried out, using AutoDock Vina [84]. The input files needed to run AutoDock Vina were protein and ligands in PDBQT format. The docking parameters of virtual screening were set up accordingly to the same control docking parameter with the RMSD value not greater than 2 Å. Virtual screening of over 4000 compounds from Natural Product Discovery (NADI) database was carried out, using the docking parameters as mentioned above. All compounds were bound similarly as the allosteric site of the crystal structure (PDB ID: 4DRI) and ranked according to the docking score.

### 3.6. Molecular Docking

From virtual screening results, we selected asiaticoside and asiatic acid from NADI and everolimus for further docking. AutoDock 4.2.6 [82] was used for this purpose, to determine a specific pose of ligand that contributes to the lowest docking score of a complex. Although the choice of AutoDock 4.2.6 was not necessarily better than AutoDock Vina [85], redocking was carried out to give further insight into the binding site from different tools. In addition, the binding sites of both FKBP5 and FRB were mostly hydrophobic; thus, AutoDock 4.2.6, which was better in discriminating the ligands in hydrophobic binding site than Autodock Vina, was used [86]. AutoDock 4.2.6 has parameters of Lamarckian Genetic Algorithm (LGA) as follows: 100 search runs, a population size of 150, elitism of 1, mutation rate of 0.02, crossover rate of 0.80, local search rate of 0.06, and energy evaluation of 2,500,000.

### 3.7. Cell Culture and Treatment

The UMB1949 cell line (ATCC^®^ CRL-4004™), derived from renal angiomyolipoma, was obtained from American Type Culture Collection (ATCC; Manassas, VA, USA). This cell line was chosen because it has a defined 5bp deletion in exon 33 of tuberin (*TSC2*) and mutations in tuberin (and/or hamartin) cause tuberous sclerosis; thus, it is a suitable target for mTOR inhibitor. The UMB1949 cell line was cultured in Dulbecco’s Modified Eagle Media (DMEM; Life Technologies, USA) supplemented with 10% of fetal bovine serum (FBS; Life Technologies, Carlsbad, CA, USA), 100 U/mL of penicillin, and 100 µg/mL streptomycin (Life Technologies, USA). The cells were incubated at 37 °C, in a humidified atmosphere containing 95% air and 5% CO_2_. The cells were seeded at a density of 5000 cells/well in 100 µL complete media and incubated for 24 h. On the following day, the complete media in each well of a 96-well plate was discarded, and the wells were washed with PBS solution. From the virtual screening, we found that compounds from *Centella asiatica* showed promising binding affinity toward mTOR; thus, they were selected for the validation study. A total volume of 100 µL of different concentrations of everolimus (10, 20, 30, 40, and 50 µM), asiaticoside (100, 200, 300, 400, and 500 µM), and asiatic acid (20, 40, 60, 80, and 100 µM) was then added into each well and incubated for 24 h, to determine the IC_50_. All drugs were obtained from Selleckchem (Houston, TX, USA).

### 3.8. Cytotoxicity Assay

A total volume of 10 µL of Cell Count Reagent SF was added into each well and incubated for 2 h, at 37 °C, in a humidified atmosphere containing 95% air and 5% CO_2_. The absorbance was measured at 450 nm, using a universal microplate reader. The experiments were carried out in triplicates. The percentage of cell viability was calculated by using the following equation [87]:

(1) $$\mathrm{Cell}\;\mathrm{viability}\;\left(\%\right)=\frac{\left(\mathrm{Absorbance}\;\mathrm{of}\;\mathrm{controlled}\;\mathrm{cells}-\mathrm{Absorbance}\;\mathrm{of}\;\mathrm{blank}\;\mathrm{cells}\right)}{\left(\mathrm{Absorbance}\;\mathrm{of}\;\mathrm{treated}\;\mathrm{cells}-\mathrm{Absorbance}\;\mathrm{of}\;\mathrm{blank}\;\mathrm{cells}\right)}$$

Absorbance of treated cells: Cells + Media + Drug concentration + DMSO.
Absorbance of controlled cells: Cells + Media + DMSO.
Absorbance of blank cells: Media.

The IC_50_ for everolimus, asiaticoside, and asiatic acid was extrapolated from the dose–response graph. The drug concentration that reduced the viability of cells by 50% (IC_50_) was determined by plotting triplicate data points over a concentration range.

### 3.9. Statistical Analysis

Analyses of the dose–response curves for everolimus, asiaticoside, and asiatic acid were performed by using the GraphPad PRISM software version 8.0.2 (GraphPad Software, Inc., San Diego, CA, USA). The IC_50_ values were expressed as mean ± SD.

## 4. Conclusions

The use of computational tools such as molecular docking and Lipinski Rule of Five filtration methods is extremely crucial and essential for the first screening evaluation in drug-discovery research. After performing in silico analysis (molecular docking) between NADI compounds against FRB domain of mTOR and FKBP5 of human protein, we selected asiaticoside and asiatic acid from *Centella asiatica* as potential mTOR inhibitors. Cytotoxicity analysis on everolimus, asiaticoside, and asiatic acid against UMB 1949 cell line for 24 h revealed that their IC_50_ was 29.5, 300, and 60 µM, respectively. As far as we are concerned, this is the first study of asiaticoside and asiatic acid as potential mTOR inhibitors for TSC disease model. These results, coupled with our in silico study, should prompt further studies to validate mTOR as the actual target and to clarify the mode of action, safety, and efficacy of these compounds as mTOR inhibitors.

## Figures and Tables

**Table 1:** Docking result of each ligand in complex with FRB and FKBP5 receptors in human.

Ligands	Docking Score (kcal/mol)	Hydrogen Bond (s)	Hydrophobic Interactions
Everolimus	−11.86	Ser2035	Tyr57, Phe67, Phe77, Trp90, Phe130, Trp2101, Tyr2038, Phe2039, Tyr2105
Asiaticoside	−11.98	Asp68, Val86, Phe2039	Phe77, Tyr113, Phe2039, Trp2101, Tyr2105, Phe2108
Asiatic acid	−10.37	Asp68	Val86, Ile87, Phe77, Phe2039, Trp2101, Tyr2105, Phe2108

## Supplementary Materials

The following are available online at https://www.mdpi.com/1420-3049/25/17/3991/s1,Table S1: The top hits from the NADI virtual screen, grouped according to the associated plants source. Table S2: Physicochemical properties of the compounds.
