# Supplementary material for: In Silico Analyses and Cytotoxicity Study of Asiaticoside and Asiatic Acid from Malaysian Plant as Potential mTOR Inhibitors
Source: Molecules. 2020 Sep 2;25(17):3991. doi: 10.3390/molecules25173991 (PMC7504803; doi:10.3390/molecules25173991)
Supplement: Supplementary file 1 [file molecules-25-03991-s001.pdf]

SUPPLEMENTARY

Table S1 The top hits from the NADI virtual screen, grouped according to the associated plants source

PLANT	NADI CODE	Docking score (kcal/mol)	COMPOUND
Endiandra kingiana	MSC3673	-13.10	Kingianin B
	MSC3676	-12.90	Kingianin E
	MSC3684	-12.80	Kingianin M
	MSC3683	-12.80	Kingianin L
	MSC3675	-12.70	Kingianin D
	MSC3678	-12.50	Kingianin G
	MSC3680	-12.30	Kingianin I
	MSC3679	-12.20	Kingianin H
	MSC3685	-11.90	Kingianin N
	MSC3681	-11.90	Kingianin J
Calophyllum inophyllum	MSC3469	-12.50	Epifriedelanol
	MSC429	-12.50	Friedelin
	MSC3471	-12.30	Canophyllol
	MSC3485	-12.20	Friedelan-3-one
	MSC3462	-12.10	Pyranojacareubin
	MSC3470	-12.00	Canophyllal
	MSC3473	-11.90	3-oxo-Friedelan-28-oic acid
	MSC3472	-11.90	Canophyllic acid
	MSC3486	-11.90	Calaustralin
Boesenbergia rotunda	MSC2076	-12.20	Panduratin F
	MSC2067	-12.20	Panduratin B1
	MSC2068	-12.10	Panduratin B2
	MSC2101	-11.90	Rotundol
	MSC2111	-11.90	(2S)-7,8-Dihydro-5-hydroxy-2-methyl-2-(4''-methyl-3''-pentenyl)-8-phenyl-2H,6H-benzo[1,2-b:5,4-b']dipyran-6-one
	MSC2075	-11.90	Panduratin E
	MSC513	-12.50	Centellasaponin D
	MSC515	-12.100	Sceffoleoside A

Centella asiatica	MSC502	-12.00	Madecassoside
	MSC514	-12.00	Asiaticoside
	MSC518	-12.00	11,12-dehydroursolic acid Lactone
Manilkara zapota	MSC3016	-14.60	Lupeol benzoate
	MSC3018	-13.00	beta-Amyrin benzoate
	MSC3020	-12.30	alpha-Spinasterol benzoate/(3b,5a,22E)-Stigmasta-7,22-dien-3-ol benzoate
	MSC3017	-12.30	beta-Amyrin acetate
	MSC3029	-12.10	Lup-20(29)-en-3.23-diol
Psidium guajava	MSC2125	-12.50	Guavacoumaric acid
	MSC2126	-12.20	Guavin B
	MSC2124	-12.20	Ilelatifol D
	MSC2136	-12.10	2alpha-Hydroxy-3beta-para-E-coumaroyloxyurs-12,18-dien-28-oic acid
	MSC2121	-11.90	Jacoumaric acid

Table S2 Physicochemical properties of the compounds

**DRUG/COMPOUND**	Everolimus	Asiaticoside	Asiatic acid
MW (g/mol)	958.2	959.12	488.699
LogP	5.9	2.33	5.44
H-Bond Donor	3	12	4
H-Bond Acceptor	14	19	5
Rotatable bond	9	10	2
Polar surface area (Å^2)	204.66	315.21	97.99
